# Unprecedented Coordination-Induced Bright Red Emission from Group 12 Metal-Bound Triarylazoimidazoles

**DOI:** 10.3390/molecules26061739

**Published:** 2021-03-20

**Authors:** Artyom A. Astafiev, Olga V. Repina, Boris S. Tupertsev, Alexey A. Nazarov, Maria R. Gonchar, Anna V. Vologzhanina, Valentine G. Nenajdenko, Andreii S. Kritchenkov, Victor N. Khrustalev, Victor N. Nadtochenko, Alexander G. Tskhovrebov

**Affiliations:** 1 N.N. Semenov Federal Research Center for Chemical Physics, Russian Academy of Sciences, Kosygina 4, 119991 Moscow, Russia; astafiev.artyom@gmail.com (A.A.A.); repina.ov@phystech.edu (O.V.R.); btoupersev@gmail.com (B.S.T.); nadtochenko@gmail.com (V.N.N.); 2 Lomonosov Moscow State University, Chemistry Department, Leninskie Gory 1/3, 119991 Moscow, Russia; alexey.nazarov@me.com (A.A.N.); mari.kainas@yandex.ru (M.R.G.); nenajdenko@gmail.com (V.G.N.); 3 Peoples’ Friendship University of Russia, Miklukho-Maklaya Street 6, 117198 Moscow, Russia; platinist@mail.ru (A.S.K.); vnkhrustalev@gmail.com (V.N.K.); 4 A.N. Nesmeyanov Institute of Organoelement Compounds, Russian Academy of Sciences, Vavilova Street 28, 119334 Moscow, Russia; vologzhanina@mail.ru; 5 N.D. Zelinsky Institute of Organic Chemistry, Russian Academy of Sciences, 47 Leninsky Prospect, 119334 Moscow, Russia

**Keywords:** azo dyes, nitrogen heterocycles, fluorescence, group 12 metal complexes

## Abstract

Arylazoimidazoles are important dyes which were intensively studied in the past. In contrast, triarylazoimidazoles (derivatives which carry aryl substituents at the imidazole core) received almost no attention in the scientific literature. Here, we report a new family of simple and easily accessible triarylazoimidazole-group 12 metal complexes, which feature highly efficient photo-luminescence emission (Φ up to  0.44). Novel compounds exhibit bright red emission in solution, which could be excited with a visible light.

## 1. Introduction

Emissive metal complexes are the key components or rapidly developing critical technologies, including organic light-emitting diodes (OLED) [1,2] photovoltaics [3,4] sensing [5,6] bioimaging [7] photodynamic therapy [8], and photocatalysis [9,10]. The progress in these areas significantly depends on the improvement of physical characteristics and their fine tuning via chemical manipulations with metal complexes’ structures [11]. Their fundamental importance and applicability stimulate active search for the new families of emissive metal complexes.

In this context, azoimidazoles are attractive chelating ligands with easily tunable electronic and photophysical properties via facile structural modifications [12,13,14,15,16,17]. Recently, we described a novel method for the synthesis of this important class of dyes, which employs nitrous oxide as a donor of azo group [17,18,19].

From the photophysics research perspective, azoimidazoles, being representatives of classical molecular switches, were comprehensively studied due to their important photochromic properties [20,21]. They are believed to be promising for applications in photopharmacology and bioinorganic chemistry since imidazole moiety is an essential group in biology, often serving as a supporting ligand in metal-containing systems [20,21]. In general, photoswitchable late transition metal complexes hold promise for the development of novel methods which would feature advantageous control of drug-action specificity [22]. Therefore, exploration of coordination chemistry of cytotoxic metals, which contain potentially photoswitchable heterocyclic ligands seemed promising.

Following our interest in azo dyes and photochromic materials [17,18,23], we turned our attention to triarylazoimidazoles and their metal complexes. Surprisingly, triarylazoimidazoles received almost no attention in the literature [24,25,26,27]. Therefore, exploration of their coordination chemistry, photophysics or photochemistry and cytotoxicity seemed as an attractive niche.

Unexpectedly, coordination of triarylazoimidazoles to group 12 metals resulted in the formation of complexes with bright red emission (quantum yields up to 44%), which could be excited with a visible light.

## 2. Results and Discussion

### 2.1. Synthesis and Structural Characterization

**Scheme 1:**
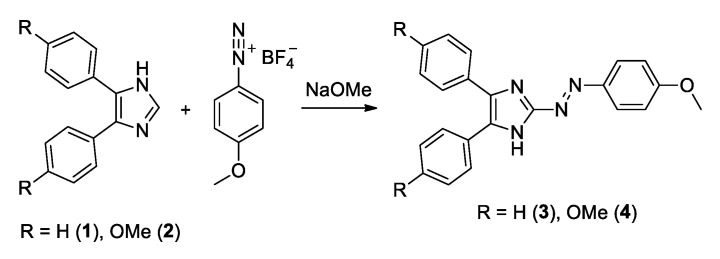
Synthesis of triarylazoimidazoles **3** and **4**.

Triarylazoimidazoles **3** and **4** were prepared via azo coupling between *p*-anisyldiazonium tetrafluoroborate and corresponding diarylimidazoles (Scheme 1) and isolated in high yields as red solids.

**Scheme 2:**
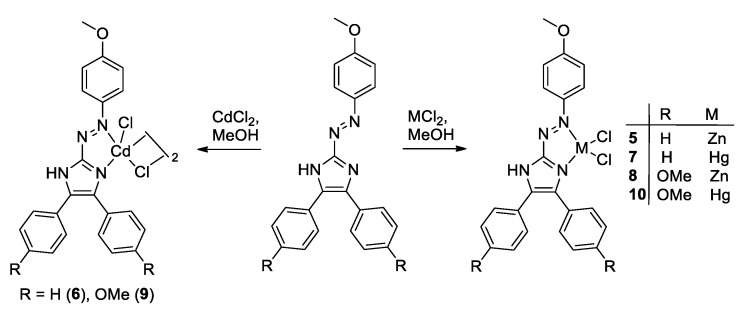
Synthesis of triarylazoimidazole complexes **5**–**10**.

When a solution of ZnCl_2_ in methanol was added to a solution of **3** in methanol the color immediately changed to dark-red and dichroic dark-green/brown microcrystalline precipitate of **5** gradually formed. Isolation and analysis of the precipitate suggested the formation of an adduct in 46% yield (Scheme 2). A similar procedure was used to form Cd and Hg complexes of **3** and Zn, Cd, and Hg complexes of **4**. The corresponding compounds **6**–**10** were isolated with yields between 43 and 71% (Scheme 2).

**Figure 1:**
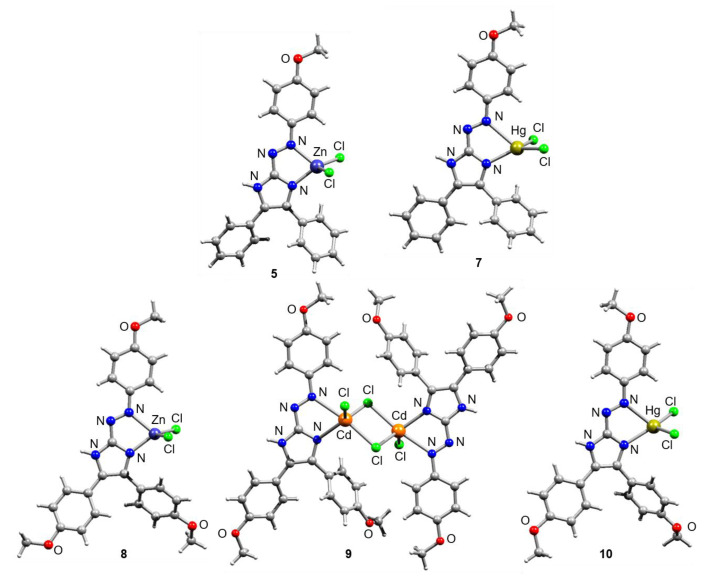
Ball-and-stick representation of the structure of **5**, **7**–**10** in the crystal. Solvent molecules are omitted. Grey and light grey spheres represent carbon and hydrogen atoms, respectively.

Complexes **5**, **7**–**10** precipitated from the reaction mixtures as well-shaped crystals, suitable for analysis by single crystal X-ray crystallography. The structural investigations confirmed the formation of Zn^II^, Cd^II^ and Hg^II^ complexes with chelating triarylazoimidazoles (Figure 1). The ligands in **5**, **7**–**10** adopt a trans configuration around the N=N bond with the azoimidazole moieties being nearly planar: dihedral N–C–N–N lie between 0.05(8)° and 2.9(6)°, while C_Ar_–C_Ar_–N–N angles do not exceed 3.6(8)°, what indicates on a significant electronic conjugation in rigid chelating triarylazoimidazoles.

Metal centers in **5**, **7**, **8**, **10** adopt distorted tetrahedral geometry (Table 1) with terminal chloride atoms, while in **9** one of two symmetrically independent chlorides act as bridging ligands so that ligands around Cd^II^ center adopt distorted trigonal bipyramidal geometry. The M–Cl and M–N bond distances increase and the N–M–N and Cl–M–Cl angles decrease with the increase in the ionic radii of Zn^II^, Hg^II^ and Cd^II^.

Structures of **5**, **7**–**10** contained one solvated methanol molecule per one metal atom, which participated in hydrogen bonding with N–H protons and chlorides (Figure S1). Interestingly, the M–Cl bond involved in hydrogen bonding was longer than the other M–Cl bond what is typical for relatively weak coordination bonds with significant ionic contribution. For **5** and **9** the hydrogen bonding resulted in the formation of infinite chains, while in **7**, **8** and **10**–hydrogen bonded tetramers were formed (Figure S1) [28,29,30,31].

### 2.2. Absorption and Emission Profiles

**Figure 2:**
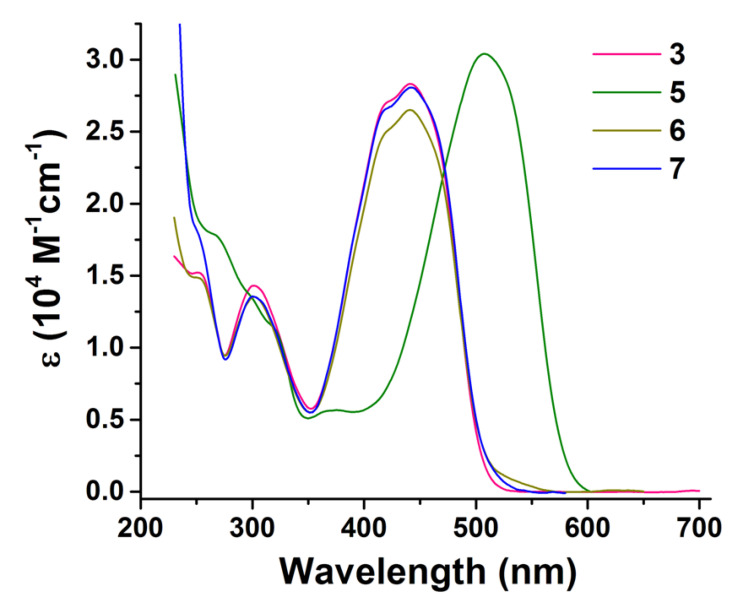
Absorption spectra of **3** and its group 12 metal complexes **5**–**7** in CH_2_Cl_2_. (ε—extinction coefficient).

The optical properties of free ligands (**3** and **4**) were quite different from their group 12 metal complexes (**5**–**10**). Electronic absorption spectra for **3** and its complexes **5**–**7** are shown in Figure 2 while for **4** and **8**–**10** in Figure S2.

Triarylazoimidazole **3** exhibited a broad absorption band with a fine structure and maximum at 441 nm, which was assigned to intraligand π-π* transitions in the azoimidazole moiety with a considerable charge transfer from azoaryl group (donor) to imidazole fragment (acceptor) [20,21]. This charge transfer band was redshifted compared to what was observed for intensively studied arylazoimidazoles which do not contain aryl substituents at the imidazole ring [20,21]. Tri(p-anisyl)azoimidazole **4** exhibited a similar blue absorption band (Figure S2), but its maximum expectedly experienced a bathochromic shift compared to that of **3**.

Coordination of **3** and **4** to group 12 metals resulted in changes in their absorption spectra (Figure 2). This effect was relatively weak for the cadmium (**6**) and mercury (**7**) derivatives, for which the main absorption band decreased slightly and a small tail appeared in the >500 nm spectral region. Binding of **4** to Hg^II^ and Cd^II^ resulted in similar changes in the absorption spectra.

Interestingly, absorption spectra of Zn^II^ complexes **5** and **8** differed dramatically from that of free ligands **3** and **4**. Absorption spectra of **5** and **8** featured a new strong band in the green spectral region which was redshifted by 67 nm (**5**) and 77 nm (**8**), compared to the absorption maxima of free ligands (Figure 2 and Figure S2). Second derivative analysis of the new peak of complex **5** (Figure S3) revealed it was heterogeneous and consisted of two overlapping peaks with maxima at ca. 498 and 536 nm, which probably corresponded to S_0_→S_2_ and S_0_→S_1_ transitions, respectively. The strongest absorption band of **8** exhibited the second derivative minimum at circa 560 nm.

We hypothesize that coordination caused a red shift of main charge transfer absorption band of the ligand and emergence of a new absorption band in the region >500 nm. That can be seen from the second derivative analysis which revealed that the main absorption peak of **5** consists of two closely situated components at 498 and 536 nm (see the SI). The first is obviously the ligand-based charge transfer transition from donating *p*-methoxyphenyl to the accepting azoimidazole group. It was red-shifted compared to the CT absorption peak of the free ligand at 441 nm due to coordination to the Zn metal center. The second component emerged as a result of coordination. Its maximum generally coincides with photoluminescence excitation maximum at 540 nm meaning that this transition populates the electronic level from which emission occurs. In complexes **6** and **7** the coordination-induced shift of CT absorption maximum was negligible and the coordination-induced component appeared as a weak absorption tail beyond 500 nm. The stronger effect of Zn coordination on absorption spectra is obviously related to the smaller size of Zn^2+^, resulting in a stronger coordination bond. The same effects (red shift of CT absorption maximum and emergence of yellow components in absorption spectra) were observed for the complexes **8**–**10**.

**Figure 3:**
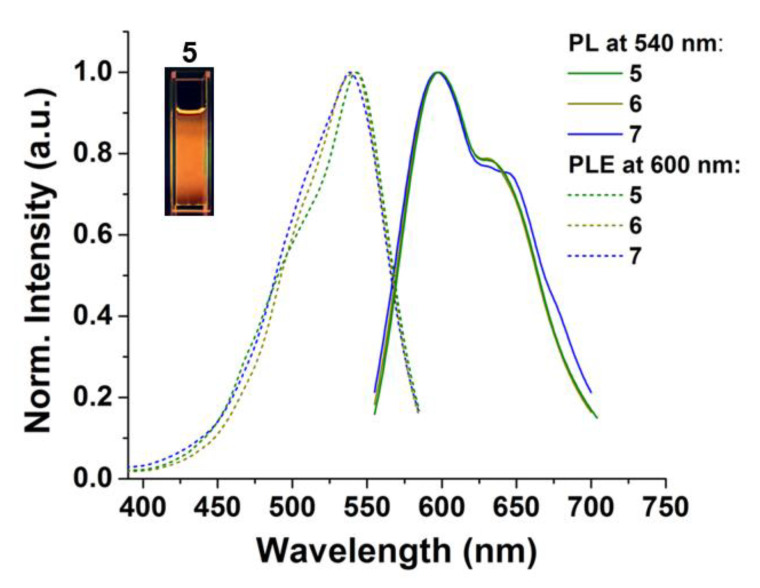
Normalized photoluminescence (PL) and photoluminescence excitation (PLE) spectra of **5**–**7** in CH_2_Cl_2_. (a.u.—arbitrary unit)

Remarkably, when a solution of **5** was subjected to white light, it showed an orange-red fluorescence seen to the naked eye while free ligand **3** exhibits no emission (even when irradiated with the UV light). Photoluminescence (PL) spectrum of **5** in CH_2_Cl_2_ exhibited a peak with emission maximum at 598 nm with a high quantum yield (39%), while photoluminescence excitation (PLE) spectrum showed a peak at 543 nm (Figure 3). A moderate Stokes shift (0.2 eV) observed between PLE and PL spectra is indicative structural differences between the ground and excited states in **5**. Normalized PL spectra of **5** did not dependent of the excitation wavelength, what was in accord with Vavilov–Kasha rule and was expected for such kind of a system.

A plausible explanation of the origin of the emission in the molecule of **5** could be a π–π* transition in a rigid coordinated triphenylazoimidazole, while in unbound azo-dye excitation could result in trans-to-cis isomerization, which serves as a relaxation channel for the excited states to decay non-radiatively. Photochemical isomerization around the N=N is intensively studied phenomenon, which was also reported for azoimidazoles [20,21]. Thus, coordination of triphenylazoimidazole at Zn(II) metal center blocks the nonradiative pathway and opens the radiative channel.

Interestingly, PL and PLE spectra of analogous Cd(II) and Hg(II) derivatives **6** and **7** qualitatively looked very similar to the Zn(II) complex **5** (Figure S1), which indicated that the origin of the emission was ligand-based, but fluorescence quantum yields of **6** and **7** (less than 9%) turned to be much smaller than that of **5**. PL spectra of **5**–**7** exhibited a shoulder at ca. 635 nm close to the main peak at ca. 600 nm, which suggested a presence of two radiative transitions of slightly different energy. PLE spectra of **5**–**7** also exhibited a shoulder at ca. 496 nm.

**Figure 4:**
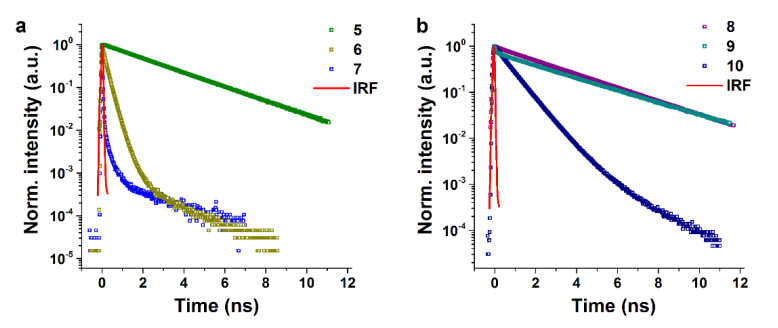
Fluorescence decay kinetics of **5**–**7** (**a**) and **8**–**10** (**b**) in CH_2_Cl_2_. Decay kinetics were monitored at 600 nm for **5**–**7** and at 660 nm for **8**–**10**. Instrumental response function (IRF) is plotted as a solid red line.

Fluorescence decay kinetics of **5** in CH_2_Cl_2_ showed single-exponential behavior with a lifetime of 2.59 ns (Figure 4). In contrast, emission decay kinetics of **6** and **7** exhibited faster and multiexponential decay, dominated by a fast decay time (Figure 4). The fast decay component for **6** was about 0.24 ns, while for **7** it was at the level of time-correlated single photon counting (TCSPC) resolution and estimated at about 20 ps from a decay kinetic deconvolution. The decrease in the fluorescence lifetime found for Zn, Cd, and Hg triad **5**–**7** was in agreement with a trend observed for the fluorescence quantum yield.

**Figure 5:**
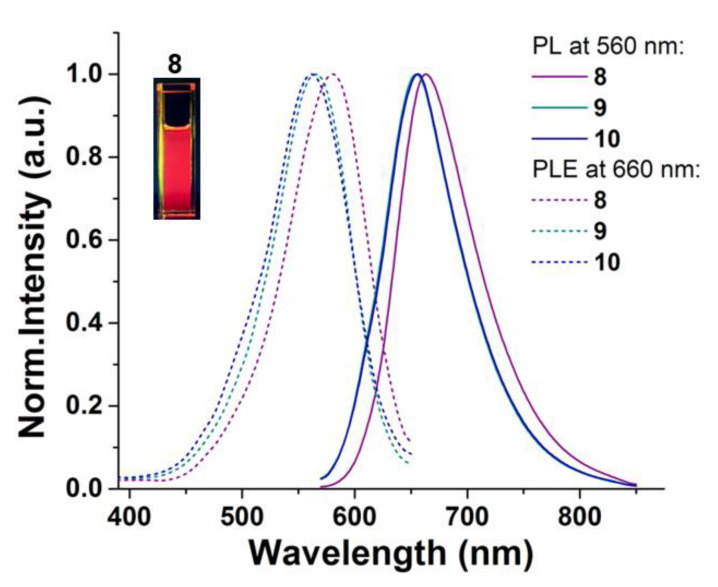
Normalized PL and PLE spectra of **8**–**10** in CH_2_Cl_2_.

PL and PLE spectra of **8**–**10** looked similar to that of **5**–**7** but featured larger Stokes shift (0.26–0.32 eV vs. 0.20–0.23 eV) (Figure 5). PL spectra of **8**–**10** showed a single broad band with a maximum at 660 nm and no fine structure. Fluorescence quantum yields for **8**–**10** (44, 39, 10%, respectively) turned out to be even higher than that of **5**–**7** (Table S2). Fluorescence decay kinetics of **8** was nearly monoexponential with a lifetime of ca. 2.9 ns (Figure 4, Table S3). Decay kinetic of **9** had a similar decay time but also a short decay component. That agrees with a smaller quantum yield of **9** in CH_2_Cl_2_ solution. Finally, photoluminescence decay of Hg complex **10** was dominated by a fast component with a decay time of 0.74 ns, the ratio of effective decay times of **8** and **10** (4:1) roughly corresponded to the ratio of quantum yields (4.5:1).

Photophysical studies for **5**–**10** in solvents of varying polarity were not successful since, in coordinating solvents, solvent molecules coordinated to azoimidazole-bound metal centers, leading to spectral changes and luminescence quantum yield which goes beyond the scope of this paper. In non-polar the complexes are virtually insoluble. PL and PLE spectra of **5** in different are given in Figure S7.

Moreover, binuclear complex **9** has generally similar photoluminescence emission and excitation spectra to mononuclear complexes **8** and **10**, so obliviously there was almost no influence on position of energy levels. Therefore, no significant photophysical differences between mononuclear and binuclear complexes were observed.

### 2.3. Cytotoxicity Evaluation

Since photoactive transition metal complexes hold promise for the development of novel methods which would feature advantageous control of drug-action specificity [22] (see Introduction), and **5**–**10** emit in near-infrared region and can be excited with a visible light, what might be useful for bioimaging, we decided to evaluate their cytotoxicity to human cancer and healthy cells. The antiproliferative activity of uncoordinated ligand **3** and its Zn^II^, Cd^II^, and Hg^II^ complexes **5**–**7** were evaluated against the human HCT116 colorectal carcinoma, MCF7 breast adenocarcinoma, A549 non-small cell lung carcinoma and WI38 nonmalignant lung fibroblast cell lines by means of standard MTT colorimetric assay as the IC_50_ value (concentration of a compound required to inhibit the cell viability by 50%) after 72 h of incubation (Table 2). Overall, cytotoxicity data showed no restriction for bioimaging applications for **5**–**10**.

Complex **3** showed moderate activity in the range of 38 to 27 µM against cancer cell lines, and no activity on the WI38 cell line. Complexes **5**–**7** displayed metal-dependent activity: Zn complex **5** showed no activity, while **6** (Cd^II^) exhibited a moderate activity and **7** (Hg^II^) found to be the most cytotoxic with antiproliferative activity close to that of cisplatin on some cell lines (Table 1).

## 3. Conclusions

In summary, we described the synthesis and characterization of group 12 metal complexes of two triarylazoimidazoles (3 and 4), which exhibit highly efficient orange-red or red photoluminescence in a solution (Φ up to  0.44). The emission can be excited with green light. Our studies highlight unexplored potential of triarylazoimidazoles for the development of a new class of emissive transition metal complexes. Further studies into synthesis of triarylazoimidazoles carrying various substituents and their metal complexes, photophysical properties, and applications are underway and will be reported in due course.

## 4. Materials and Methods

General remarks. Unless stated otherwise, all the reagents used in this study were obtained from the commercial sources (Aldrich, TCI-Europe, Strem, ABCR). NMR spectra were recorded on a Bruker Avance III, Karlsruhe, Germany (^1^H: 400 MHz); chemical shifts (*δ*) are given in ppm relative to TMS, coupling constants (J) in Hz. The solvent signals were used as references (CDCl_3_: *δ*_C_ = 77.16 ppm; residual CHCl_3_ in CDCl_3_: *δ*_H_ = 7.26 ppm; CD_2_Cl_2_: *δ*_C_ = 53.84 ppm; residual CHDCl_2_ in CD_2_Cl_2_: *δ*_H_ = 5.32 ppm); ^1^H and ^13^C assignments were established using NOESY, HSQC, and HMBC experiments; numbering schemes as shown in the Inserts. IR: Perkin–Elmer Spectrum One spectrometer, wavenumbers (*ṽ*) in cm^−1^. C, H, and N elemental analyses were carried out on a Euro EA 3028HT CHNS/O analyzer (Pavia, Italy). Mass-spectra were obtained on a Bruker micrOTOF spectrometer equipped with electrospray ionization (ESI) source (Bremen, Germany); MeOH, CH_2_Cl_2_, or MeOH/CH_2_Cl_2_ mixture was used as a solvent. Thermogravimetric analysis (TGA) and differential thermal analysis were determined using a Netzsch TG 209F1 Libra apparatus (Selb, Germany). Solvents were purified by distillation over the indicated drying agents and were transferred under Ar: Et_2_O (Mg/anthracene), CH_2_Cl_2_ (CaH_2_), hexane (Na/K). Flash chromatography: Merck Geduran® Si 60 (40–63 μm). Absorption spectra were measured in a 4 mL quartz cuvette using a UV-VIS spectrometer (UV-3600, Shimadzu, Kyoto, Japan) and luminescence emission and excitation spectra in the same cuvette — using a spectrofluorimeter (RF-5031PC, Shimadzu, Kyoto, Japan). The luminescence quantum yield was determined using the slope method relative to the reference fluorophore, which was the ethanol solution of rhodamine B (Φ = 0.68) excited at 540 nm for the complexes **5**–**7** and the ethanol solution of Nile Blue due (Φ = 0.17) excited at 560 nm for complexes **8**–**10**, using a series of ethanol solutions of the sample with varying concentrations. The luminescence lifetime was measured using frequency-doubled pulses of a femtosecond titanium–sapphire oscillator (Tsunami, Spectra-Physics, Santa Clara, CA, USA) with a central wavelength of 490 nm, repetition rate of 80 MHz, duration of 100 fs, and pulse energy of 10 pJ. After passing through a FESH0750 dielectric filter (Thorlabs, Newton, NJ, USA) mounted at an angle of 45°, femtosecond laser pulses were coupled into an objective lens (Olympus, 20×, 0.55 NA) and focused into a penicillin vial with a sample. Luminescence was collected by the same lens, filtered by a long-pass filter (FELH0500, Thorlabs), and directed to a monochromator (Acton SP300i, Sarasota, FL, USA), where it was detected by the photomultiplier tube of a time-correlated photon counting system (SPC-150N, Becker and Hickl GmbH, Berlin, Germany), which recorded the luminescence decay kinetics in the time range of 0–12.5 ns with a resolution of 20 ps. Decay kinetics were monitored at the emission wavelength of 600 nm for **5**–**7** and 660 nm-for **8**–**10**. Instrumental response function (IRF) was measured at 490 nm using reflection of the laser beam from a coverslip surface. Parameters of mono- and multiexponential decay were found by deconvolution of the measured decay kinetics using the SPCImage 8.1 software (Becker and Hickl GmbH, Berlin, Germany) and registered IRF. Luminescence decay kinetics were fit with either a mono- or multiexponential decay function using the SPCImage software (Becker and Hickl GmbH). Coefficients of the multiexponential fit A_i_ were normalized so that $\underset{i}{\sum }A_{i}=1$. Effective decay time T_eff_ was calculated as $T_{eff}=\;\underset{i}{\sum }A_{i}T_{i}$.

General procedure for the synthesis of triarylazoimidazoles **3** and **4**. Methanol solution of NaOMe (30 wt %, 1 equivalent, for the quantity see below) and a solution of *p*-anisyldiazonium tetrafluoroborate (1 equivalent) in 10 mL MeCN and were sequentially added to a solution of diarylimidazole 1 or 2 in MeOH (25 mL). The resulting mixture stirred for 1 h, evaporated, redissolved in CH_2_Cl_2_, filtered, evaporated again, washed with Et_2_O (3 × 3 mL), and dried under vacuum.

**3.***p*-anisyldiazonium tetrafluoroborate (2.270 mmol, 504 mg), 4,5-bis-(phenyl)imidazole (2.270 mmol, 500 mg) and NaOMe (2.270 mmol, 425 µL) were used. Yield: 748 mg (93%). Elem. anal. calcd for C_22_H_18_N_4_O: C 74.56; H 5.12; N 15.81. Found: C 74.89; H 5.62; N 15.41. ^1^H NMR (400 MHz, DMSO-*d6*): *δ* 7.91(d, *J* = 9.1 Hz, 2H), 7.31–7.47 (m, 10H), 7.16 (d, *J* = 9.1 Hz, 2H), 3.88 (s, 3H). NH proton was not observed due to the exchange with DMSO-*d6*. ^13^C{^1^H} NMR (151 MHz, DMSO-*d6*): *δ* 209.9, 162.2, 153.9, 146.5, 134.9, 129.0, 128.5, 124.5, 114.9, 55.7. MS (ESI^+^), found: 355.1553 [M + H]^+^; calcd for C_22_H_19_N_4_O: 355.1556. UV/Vis (CH_2_Cl_2_): *λ*_max_ = 441 nm, *ε* = 2.83 × 10^4^ M^−1^cm^−1^.

**4.***p*-anisyldiazonium tetrafluoroborate (1.784 mmol, 396 mg), 4,5-bis-(*p*-anisyl)imidazole (1.784 mmol, 500 mg) and NaOMe (1.784 mmol, 335 µL) were used. Yield: 658 mg (89%). Elem. anal. calcd for C_24_H_22_N_4_O_3_: C 69.55; H 5.35; N 13.52. Found: C 69.81; H 5.76; N 13.18. ^1^H NMR (400 MHz, DMSO-*d6*): *δ* 7.80 (d, *J* = 9.0 Hz, 2H), 7.42 (d, *J* = 8.8 Hz, 4H), 7.07 (d, *J* = 9.0 Hz, 2H), 6.89 (d, *J* = 8.8 Hz, 4H), 3.82 (s, 3H), 3.74 (s, 6H). NH proton was not observed due to the exchange with DMSO-*d6*. ^13^C{^1^H} NMR (151 MHz, DMSO-*d6*): *δ* 206.5, 161.1, 158.4, 147.1, 129.1, 123.8, 114.6, 113.7, 55.6, 55.1. MS (ESI^+^), found: 415.1776 [M + H]^+^; calcd for C_24_H_23_N_4_O_3_: 415.1770. UV/Vis (CH_2_Cl_2_): *λ*_max_ = 465 nm, *ε* = 2.24 × 10^4^ M^−1^cm^−1^.

General procedure for the synthesis of **5**–**10**. Methanol solution (1 mL) of 3 or 4 (1 equivalent, for the quantity see below) was added to a solution of an appropriate metal halide (1 equivalent) in 1 mL MeOH (1 mL). The resulting mixture was kept without stirring for 24 h, the formed precipitate was filtered, washed with MeOH (3 × 1 mL), Et_2_O (3 × 3 mL), and dried under vacuum.

**5.** Complex **3** (0.141 mmol, 50 mg), ZnCl_2_ (0.141 mmol, 19 mg) were used. Yield: 32 mg (46%). Elem. anal. calcd for C_22_H_18_Cl_2_N_4_OZn: C 53.85; H 3.70; N 11.42. Found: C 54.16; H 4.04; N 10.98. ^1^H and ^13^C{^1^H} NMR spectra were not obtained due to the low solubility of the compound in the common deuterated solvents. Crystals, suitable for X-ray analysis, were obtained from the reaction mixture. UV/Vis (CH_2_Cl_2_): *λ*_max_ = 508 nm, *ε* = 3.04 × 10^4^ M^−1^cm^−1^.

**6.** Complex **3** (0.141 mmol, 50 mg), CdCl_2_ 2H_2_O (0.141 mmol, 26 mg) were used. Yield: 40 mg (53%). Elem. anal. calcd for C_44_H_36_Cd_2_Cl_4_N_8_O_2_: C 49.14; H 3.37; N 10.42. Found: C 49.56; H 3.74; N 10.11. ^1^H and ^13^C{^1^H} NMR spectra were not obtained due to the low solubility of the compound in the common deuterated solvents. Crystals, suitable for X-ray analysis, were obtained from the reaction mixture. UV/Vis (CH_2_Cl_2_): *λ*_max_ = 441 nm, *ε* = 2.65 × 10^4^ M^−1^cm^−1^.

**7.** Complex **3** (0.141 mmol, 50 mg), HgCl_2_ (0.141 mmol, 38 mg) were used. Yield: 60 mg (68%). Elem. anal. calcd for C_22_H_18_Cl_2_HgN_4_O: C 42.22; H 2.90; N 8.95. Found: C 42.64; H 3.28; N 8.68. ^1^H and ^13^C{^1^H} NMR spectra were not obtained due to the low solubility of the compound in the common deuterated solvents. Crystals, suitable for X-ray analysis, were obtained from the reaction mixture. UV/Vis (CH_2_Cl_2_): *λ*_max_ = 442 nm, *ε* = 2.81 × 10^4^ M^−1^cm^−1^.

**8.** Complex **4** (0.121 mmol, 50 mg), ZnCl_2_ (0.121 mmol, 16.5 mg) were used. Yield: 38 mg (57%). Elem. anal. calcd for C_24_H_22_Cl_2_ZnN_4_O_3_: C 52.34; H 4.03; N 10.17. Found: C 52.56; H 4.43; N 9.78. ^1^H and ^13^C{^1^H} NMR spectra were not obtained due to the low solubility of the compound in the common deuterated solvents. Crystals, suitable for X-ray analysis, were obtained from the reaction mixture. UV/Vis (CH_2_Cl_2_): *λ*_max_ = 542 nm, *ε* = 2.72 × 10^4^ M^−1^cm^−1^.

**9.** Complex **4** (0.121 mmol, 50 mg), CdCl_2_ 2H_2_O (0.121 mmol, 22 mg) were used. Yield: 31 mg (43%). Elem. anal. calcd for C_48_H_44_Cd_2_Cl_4_N_8_O_6_: C 48.22; H 3.71; N 9.37. Found: C 48.58; H 3.35; N 8.89. ^1^H and ^13^C{^1^H} NMR spectra were not obtained due to the low solubility of the compound in the common deuterated solvents. Crystals, suitable for X-ray analysis, were obtained from the reaction mixture. UV/Vis (CH_2_Cl_2_): *λ*_max_ = 479 nm, *ε* = 1.90 × 10^4^ M^−1^cm^−1^.

**10.** Complex **4** (0.121 mmol, 50 mg), HgCl_2_ (0.121 mmol, 33 mg) were used. Yield: 59 mg (71%). Elem. anal. calcd for C_24_H_22_Cl_2_HgN_4_O_3_: C 42.02; H 3.23; N 8.17. Found: C 42.28; H 3.13; N 7.73. ^1^H and ^13^C{^1^H} NMR spectra were not obtained due to the low solubility of the compound in the common deuterated solvents. Crystals, suitable for X-ray analysis, were obtained from the reaction mixture. UV/Vis (CH_2_Cl_2_): *λ*_max_ = 474 nm, *ε* = 2.06 × 10^4^ M^−1^cm^−1^.

X-ray diffraction studies. Single crystals of 5·CH_3_OH, 7·CH_3_OH, 8·CH_3_OH, 9·2CH_3_OH, and 10·CH_3_OH were obtained from reaction mixtures. XRD data were collected using equipment of the Center for Molecular Studies of INEOS RAS. Intensities of the reflections for these crystals were collected with Bruker Apex II Duo CCD diffractometer (for 5·CH_3_OH, 7·CH_3_OH, 8·CH_3_OH, and 10·CH_3_OH) at 120.0(2) K and with Bruker Quest diffractometer with PHOTON detector (for 9·2CH_3_OH) at room temperature (MoKα-radiation, λ = 0.71073 Å). The structures were solved by the SHELXT method [32] and refined by full-matrix least squares against *F*^2^. Non-hydrogen atoms were refined anisotropically except for a disordered methoxyphenyl fragment of 9·2CH_3_OH. The methoxyphenyl is equally disordered over two sites, and non-hydrogen atoms for this fragment were refined isotropically H(N) and H(O) atoms were located on difference Fourier maps, and those of H(C) atoms were calculated. All hydrogen atoms were included in a refinement by the riding model with *U_iso_*(H) = 1.5*U_eq_*(X) for methyl and hydroxy groups and 1.2*U_eq_*(X) for the other atoms. All calculations were made using the SHELXL2014 [33] and OLEX2 [34] program packages. Crystallographic parameters and refinement details for all complexes are listed in Table S1. Overall, metrical parameters for triarylazoimidazoles in 5, **7**–**10** are similar to those reported for structurally relevant azocompounds [17,18,19,23,35,36] and imidazole derivatives [37,38]. CCDC 2058877-2058881 contain the supplementary crystallographic data for this paper. These data can be obtained free of charge via http://www.ccdc.cam.ac.uk/structures/ (accessed 17 March 2021).

## Figures and Tables

**Table 1:** Selected geometrical parameters (Å, °) for **5**, **7**–**10**.

	5·CH_3_OH	8·CH_3_OH	7·CH_3_OH	10·CH_3_OH	9·2CH_3_OH
M	Zn^II^	Zn^II^	Hg^II^	Hg^II^	Cd^II^
M–Cl	2.1929(8)–2.2300(8)	2.1852(4)–2.2401(4)	2.337(1)–2.493(1)	2.366(2)–2.477(2)	2.441(2)–2.629(1)
M–N1_Im_	2.023(2)	2.023(1)	2.189(3)	2.224(5)	2.252(3)
M–N4_azo_	2.151(2)	2.147(1)	2.536(3)	2.501(6)	2.551(3)
N1_Im_–M–N4_azo_	78.2(1)	77.83(5)	68.0(1)	69.9(2)	68.5(1)
Cl–M–Cl	117.43(3)	119.09(2)	111.51(4)	114.22(7)	89.05(5)–121.45(5)
Cl1–M–N	113.0(1)–122.0(1)	116.24(3)–118.09(4)	99.91(9)–105.8(1)	113.2(2)–136.2(2)	97.8(1)–118.6(1)
Cl2–M–N	108.82(6)–110.36(7)	106.57(4)–111.49(4)	122.6(1)–137.5(1)	104.9(2)–106.1(2)	85.43(8)–116.7(1)
N1–C	1.371(3)	1.371(2)	1.365(5)	1.368(8)	1.366(5)
N1=C	1.339(3)	1.335(1)	1.325(5)	1.335(9)	1.330(5)
C–N(H)	1.341(3)	1.346(2)	1.349(5)	1.344(8)	1.338(5)
(Ar)C–N1_azo_	1.388(3)	1.381(2)	1.391(5)	1.394(8)	1.387(4)
N(H)–C(Ar)	1.372(3)	1.378(2)	1.375(5)	1.381(8)	1.372(4)
(Ar)C=C(Ar)	1.396(4)	1.405(2)	1.392(5)	1.398(9)	1.386(5)
N=N	1.286(3)	1.287(2)	1.270(5)	1.276(7)	1.273(4)
Ar–C	1.473(4)–1.478(4)	1.461(2)–1.476(2)	1.464(4)–1.482(5)	1.459(8)–1.475(9)	1.465(5)–1.513(5)
Ar–N_azo_	1.404(3)	1.398(2)	1.400(5)	1.398(8)	1.406(4)

**Table 2:** Cytotoxicity (IC_50_, μM) of **3** and **5**–**7** following incubation for 72 h with human HCT116 colorectal carcinoma, MCF7 breast adenocarcinoma, A549 non-small cell lung carcinoma and WI38 nonmalignant lung fibroblast cell lines.

	HCT116	MCF7	A549	WI38
**3**	38.8 ± 0.4	27.3 ± 1.5	33.5 ± 5.3	>100
**5**	>100	>100	>100	>100
**5**	>100	>100	>100	>100
**6**	>100	42.8 ± 0.1	17.1 ± 3.5	53.5 ± 2.0
**7**	10.4 ± 2.4	14.2 ± 2.5	30.0 ± 3.3	6.4 ± 1.3
Cisplatin	12.3 ± 1.7	12.5 ± 1.2	8.8 ± 0.9	3.0 ± 0.7

## Supplementary Materials

The following are available online. Table S1. Crystallographic data and the refinement parameters for the crystals of **5**, **7**–**10**. Figure S1. H-bonded architectures in **5**, **7** and **9**. Figure S2. UV-VIS spectra of **4** and its group 12 metal complexes **8**–**10** in CH_2_Cl_2_. Figure S3. UV-VIS spectrum of **5** and its second derivative. Figure S4**.** PLE spectrum of **5** and its second derivative. Figure S5. UV-VIS spectrum of **8** and its second derivative. Figure S6. PLE spectrum of **8** and its second derivative. Table S2. Photophysical properties of ligands **3** and **4** and their complexes **5**–**10** in CH_2_Cl_2_. Table S3. Parameters of multiexponential fit of photoluminescence decay kinetics of complexes **5**–**7** and **8**–**10** in CH_2_Cl_2_. Figure S7. PL and PLE spectra of **5** in ethanol, acetonitrile, dichloromethane and toluene.

## Sample Availability

Samples of the compounds **3**–**10** are available from the authors.
