# Supplementary material for: Unprecedented Coordination-Induced Bright Red Emission from Group 12 Metal-Bound Triarylazoimidazoles
Source: Molecules. 2021 Mar 20;26(6):1739. doi: 10.3390/molecules26061739 (PMC8003801; doi:10.3390/molecules26061739)
Supplement: Supplementary file 1 [file molecules-26-01739-s001.pdf]

Supporting Information

Unprecedented Coordination-induced Bright Red Emission from Group 12 Metal-bound Triarylazoimidazoles

Artyom A. Astafiev,[1,2] Olga V. Repina,[1] Boris S. Tupertsev,[1] Alexey A. Nazarov,[2] Maria R. Gonchar,[2] Anna V. Vologzhanina,[3] Valentine G. Nenajdenko,[2] Andreii S. Kritchenkov,[4] Victor N. Khrustalev,[4,5] Victor N. Nadtochenko,[1,2] and Alexander G. Tskhovrebov[1,4]*

1 N.N. Semenov Federal Research Center for Chemical Physics, Russian Academy of Sciences, Ul. Kosygina 4, Moscow, Russian Federation

2 Lomonosov Moscow State University, Leninskie Gory 1/3, Moscow, Russian Federation

3 A.N. Nesmeyanov Institute of Organoelement Compounds, Russian Academy of Sciences, Vavilova str. 28, Moscow, Russian Federation

4 Peoples' Friendship University of Russia, Miklukho-Maklaya Str. 6, Moscow, Russian Federation

5 N.D. Zelinsky Institute of Organic Chemistry, Russian Academy of Sciences, 47 Leninsky Prosp., Moscow, Russian Federation

Table S1. Crystallographic data and the refinement parameters for the crystals of **5**, **7–10**.

	**5** CH$_3$OH	**7** CH$_3$OH	**8** CH$_3$OH	**9** 2CH$_3$OH	**10** CH$_3$OH
Empirical formula	$C_{23}H_{22}Cl_2N_4O_2Zn$	$C_{23}H_{22}Cl_2HgN_4O_2$	$C_{25}H_{26}Cl_2N_4O_4Zn$	$C_{50}H_{52}Cd_2Cl_4N_8O_8$	$C_{25}H_{26}Cl_2HgN_4O_4$
Fw	522.71	657.93	582.77	1259.59	717.99
Crystal system	Monoclinic	Triclinic	Triclinic	Triclinic	Monoclinic
Space group	$P2_1/n$	$P\bar{1}$	$P\bar{1}$	$P\bar{1}$	$P2_1/c$
Z	4	2	2	1	4
a, Å	14.4265(15)	9.1793(15)	9.3141(6)	9.6763(9)	9.0019(18)
b, Å	10.0183(10)	12.0859(16)	11.4977(7)	10.9199(9)	19.616(4)
c, Å	16.7355(17)	12.2758(15)	13.9953(8)	14.2076(12)	15.206(3)
α, °	90	105.774(2)	109.5870(10)	109.931(3)	90
β, °	102.443(2)	105.588(2)	104.9620(10)	97.972(4)	103.958(5)
γ, °	90	101.724(2)	96.0060(10)	102.717(4)	90
V, Å^3	2362.0(4)	1205.0(3)	1333.73(14)	1338.8(2)	2605.8(9)
D_{calc}, g·cm^{-3}	1.470	1.813	1.451	1.562	1.830
μ, cm^{-1}	1.293	6.635	1.159	1.053	6.151
F(000)	1072	636	600	636	1400
No of measured reflections	26124	21780	21846	24991	29996
No of independent reflections	7432	10884	10200	7583	7979
R_{int}	0.118	0.043	0.026	0.073	0.106
No of observed reflections with [I>2σ(I)]	4366	7555	7727	4246	5177
No of parameters	292	292	329	309	330
R_1 [I>2σ(I)]	0.0549	0.0512	0.0334	0.0515	0.0542
wR_2 [all]	0.0878	0.1302	0.0814	0.1269	0.1397
GOF	0.989	0.985	0.950	1.045	1.016
$\Delta\rho_{max}$, $\Delta\rho_{min}$ (e Å^{-3})	0.562/-0.914	4.242/-4.150	0.571/-0.409	0.405/-0.811	3.016/-3.133

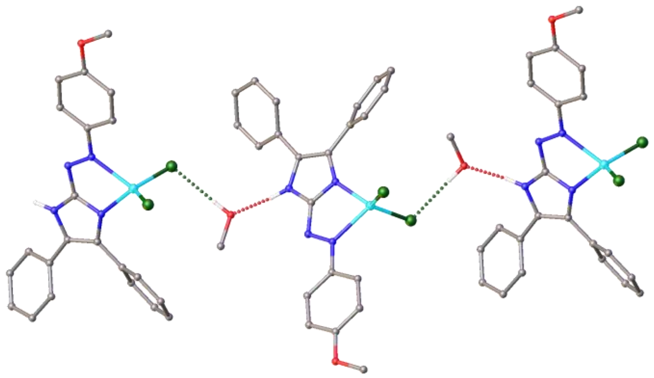

5

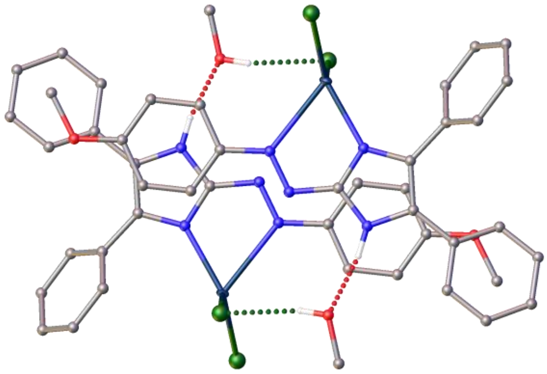

7

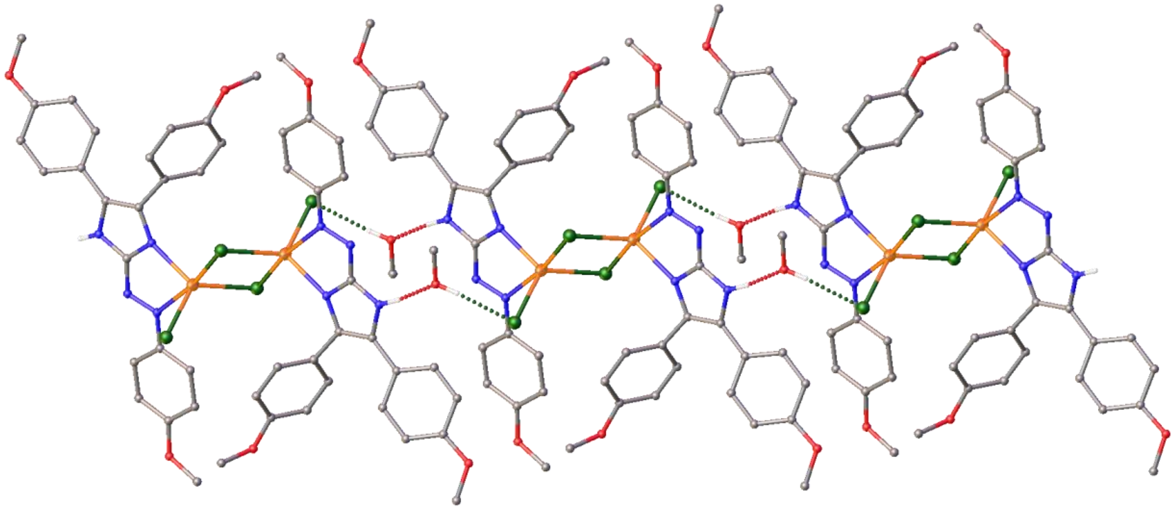

9

Figure S1. H-bonded architectures in **5**, **7** and **9**. Color code: C - grey, Cd - orange, Cl - green, H - white, Hg - yellow, N - blue, O - red, Zn – light blue. The H(C) atoms are omitted. H-bonds are dotted.

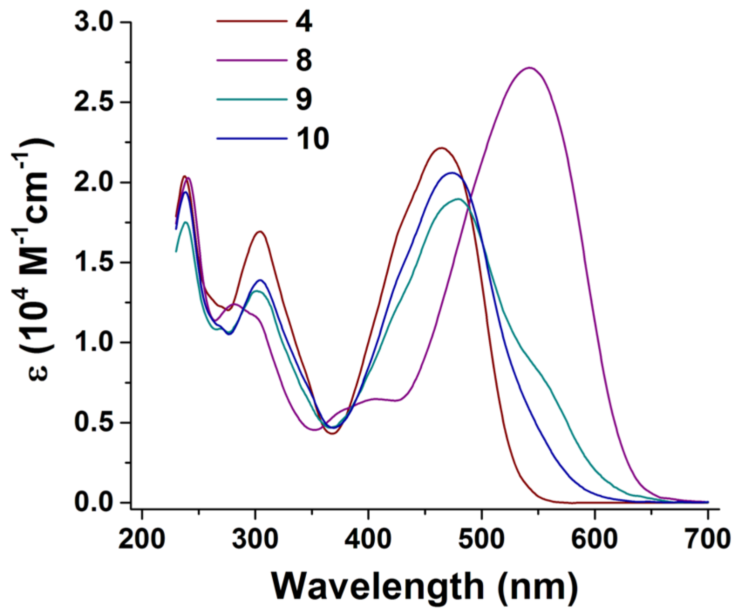

Figure S2. UV-VIS spectra of **4** and its group 12 metal complexes **8–10** in CH_2Cl_2.

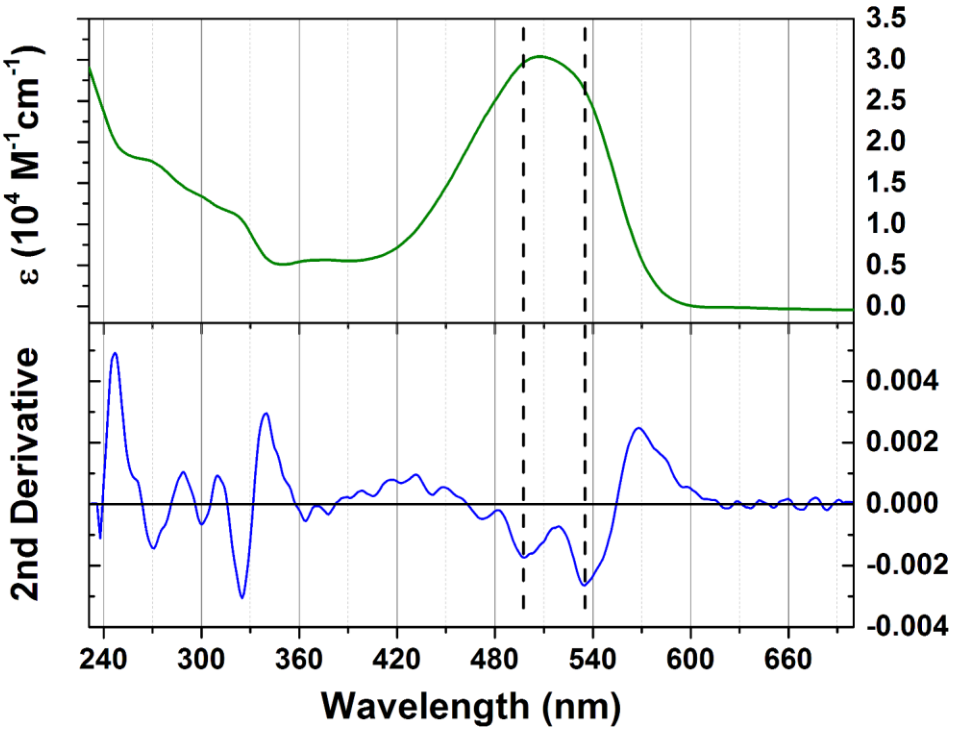

Figure S3. UV-VIS spectrum of **5** and its second derivative.

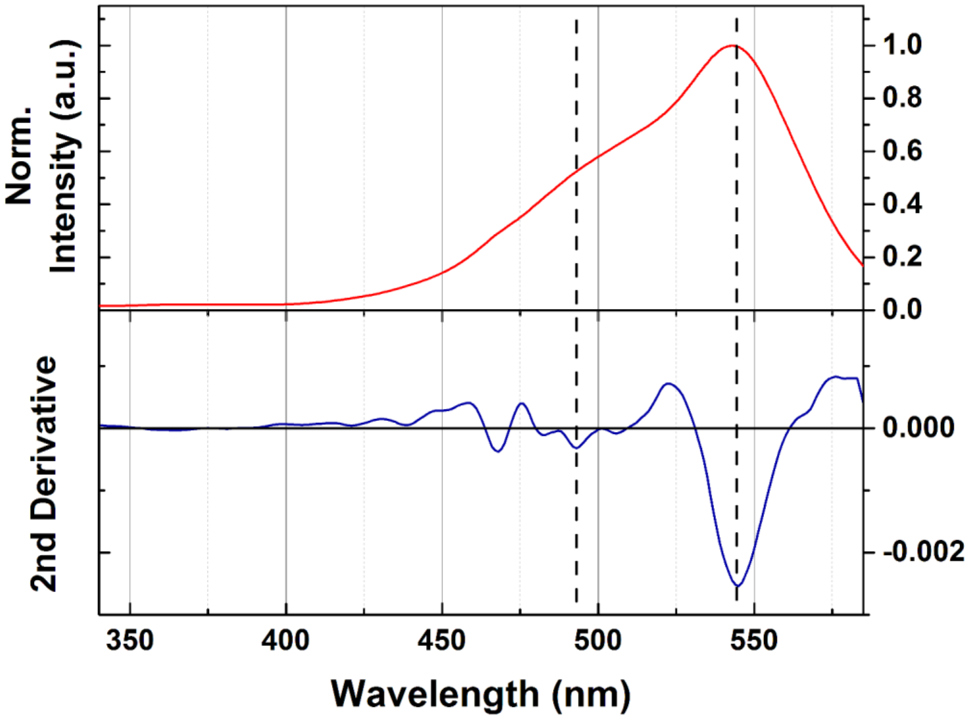

Figure S4. PLE spectrum of **5** and its second derivative.

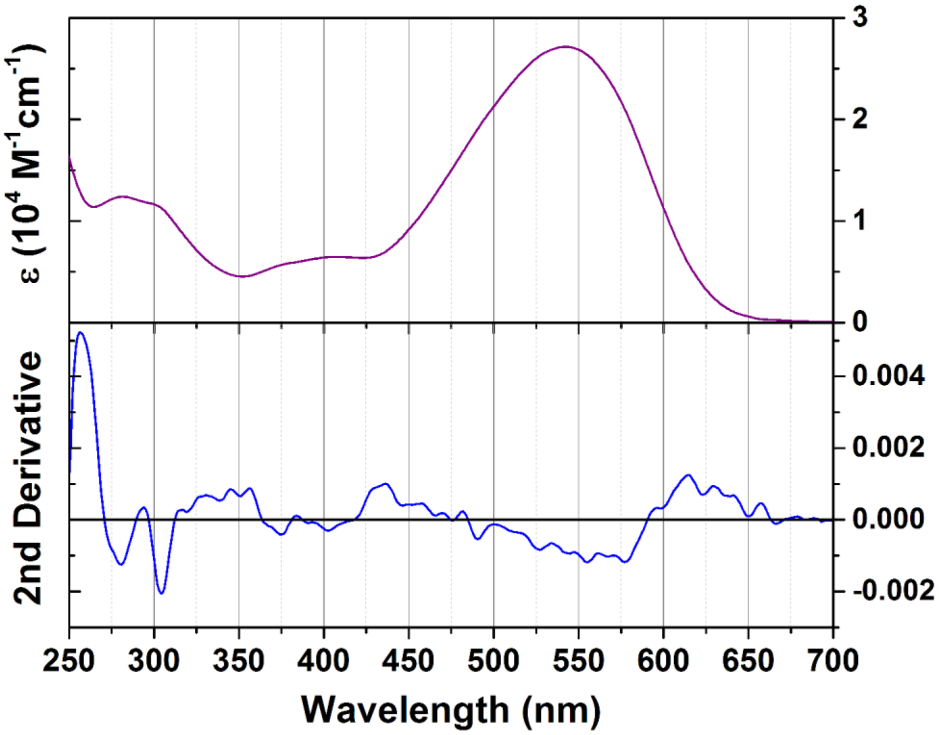

Figure S5. UV-VIS spectrum of **8** and its second derivative.

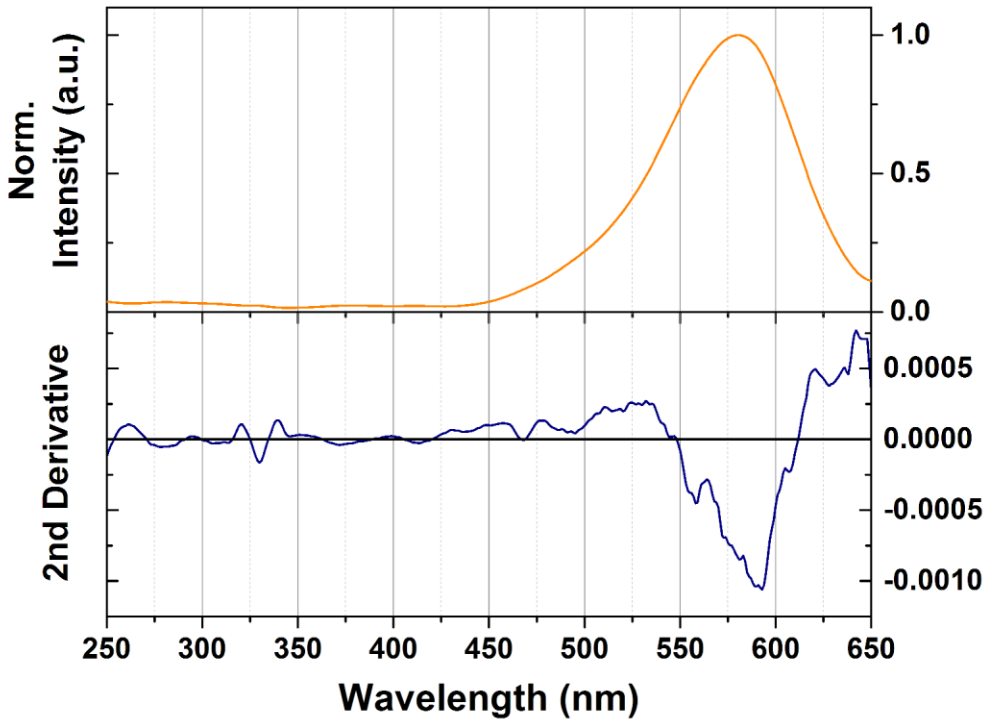

Figure S6. PLE spectrum of **8** and its second derivative.

Table S2. Photophysical properties of ligands **3** and **4** and their complexes **5–10** in CH_2Cl_2. N.D. – not determined.

	λ_{max}, abs [nm]	ε_{max} [$M^{-1}cm^{-1}$]	λ_{max}, ex [nm]	λ_{max}, em [nm]	Φ_{em}
**3**	441	28320	N.D.	N.D.	N.D.
**4**	465	22410	N.D.	N.D.	N.D.
**5**	508	30410	543	598	0.391
**6**	441	26510	542	598	0.088
**7**	442	28070	538	597	<0.08
**8**	542	27150	581	663	0.440
**9**	479	18960	565	655	0.389
**10**	474	20590	562	656	0.097

Unlike **5** complexes **6** and **7** have only small absorption at the photoluminescence excitation maximum (ca. 540 nm) and their luminescence quantum yields are also smaller. Generally, we observed a correlation between the emission quantum yield and the strength of absorption in the yellow spectral region. Both emission quantum yield and absorption intensity followed the

trend Zn>Cd>Hg which was an inverse trend of the ion radius. There was also a correlation with Me-N bond length in the complexes which for Zn was smaller than for Cd and Hg (**Table 1**). That suggests that the strength of the coordination bond affected the rate of nonradiative relaxation and Zn complexes **5** and **8** with stronger coordination bonds had the smallest nonradiative rate and the largest quantum yield.

Table S3. Parameters of multiexponential fit of photoluminescence decay kinetics of complexes **5**–**7** and **8**–**10** in CH_2Cl_2. Decay kinetics of photoluminescence were monitored at 600 nm for **5**–**7** and at 660 nm for **8**–**10**.

	A_1	T_1, ns	A_2	T_2, ns	A_3	T_3, ns	T_{eff}, ns
**5**	1	2.59					2.59
**6**	0.98	0.24	0.02	2.21			0.28
**7**	0.991	0.02	0.008	0.233	0.001	2.32	0.024
**8**	1	2.92					2.92
**9**	0.262	0.06	0.738	3.03			2.25
**10**	0.948	0.74	0.052	1.40			0.77

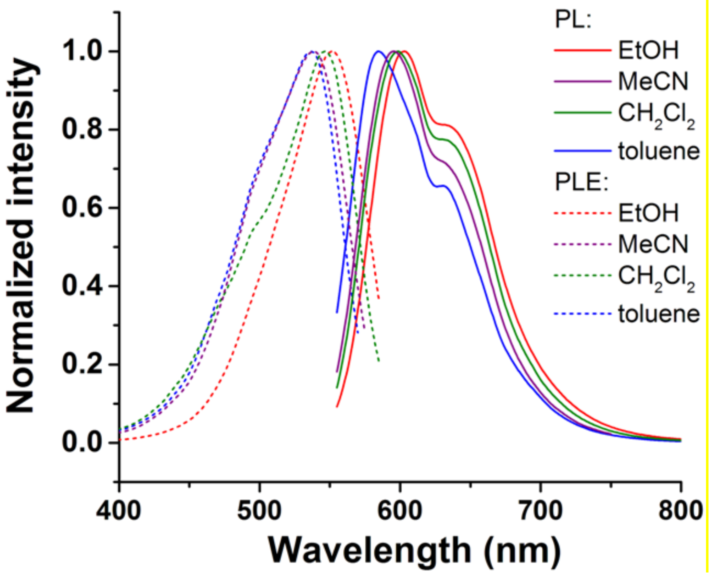

Figure S7. PL and PLE spectra of **5** in ethanol, acetonitrile, dichloromethane and toluene.
